# Round about the Asylums, XIV

**Published:** 1888-10-13

**Authors:** 


### Round About the Asylums.—XIV.

By a Medical Superintendent.

*(Concluded from p. 336, Vol. IV.)*

### SUMMARY.

It would be undesirable to review the annual reports of all the asylums and lunatic hospitals, but it may be assumed that those we have noticed are fair representatives of their respective classes, as there was no selection made. We believe, therefore, that the readers of The Hospital have been presented with samples whereby the mass may be judged.

In most of our notices we have given the number of patients in the asylums, the percentages of recoveries, and the percentages of deaths, together with any point which seemed to call for special mention, or in which the asylum was different from its fellows.

The statistics of one year are not sufficient to draw any reliable conclusion from, either as to the value of the institution as a hospital for the cure of disease or as an asylum for the care of chronic lunatics. Even the collected statistics of a number of years would not teach us much, because the conditions are not the same in any two asylums, or in the same asylum at different times. For instance, one asylum will have fewer than the average of paralytics and epileptics, another will have more than a fair share, a third will draw its patients from a purely urban population, a fourth chiefly from an agricultural district, and so on.

It would seem that the number of the insane in England and Wales on the 1st of January, 1888, was 82,643, being at the rate of 28·87 per 10,000 of the population, whereas in 1859 there were only 36,762, being at the rate of 18·67 per 10,000.

The percentage of recoveries last year was 38·56 on the admissions, which is a trifle under the average for the last ten years. The percentage of deaths, calculated on the average number resident, was 9·56, being also a little under the average.

Three thousand four hundred and thirty-seven suicidal pauper cases were admitted during the year, and 507 private cases during the same period. Nineteen of them committed suicide, but only twelve of these were in county asylums, the remaining seven being in lunatic hospitals or in licensed houses—a fact which seems to show that in this particular, as in so many others, the lunatic pauper is better off than the private patient.

Not a few medical superintendents have of late years commented on the injustice which the Home Office inflicts on our county and borough asylums by sending criminal lunatics to them under warrant. At the close of 1887 there were 119 criminals in our public asylums, and 55 criminals were during the year discharged absolutely as criminals, but retained as ordinary pauper lunatics, making a total of 174. Even this number, large as it looks, is within the mark, as there must be many cases hanging on from previous years. It is really quite time that some steps were taken to free our county asylums from these irreconcilables, whose presence entails much anxiety on the staff, and much injury to the other inmates.

We have said that the percentage of recoveries was not so high as in the previous year; but we must not assume that fewer cases are cured now than formerly. In fact, just the opposite is true. There is not much doubt that the percentage of recoveries in recoverable cases is higher than it ever was before; but more chronic and imbecile patients are now sent to our asylums, and these being reckoned with the others in all our statistics, the percentage is reduced.

When the Royal Commission on the Lunacy Laws was taking evidence, one of the witnesses proposed that acute cases should be treated in separate hospitals; and lately a leader in the *Times* has reintroduced the subject, suggesting the erection of these Acute Hospitals, and proposing the appointment of visiting physicians similar to the general hospitals. The latter idea could only have entered into the head of a man who wanted to be made a visiting physician himself, and so obtain a secure footing in the specialty, while the advantages which the hospital itself would have over the acute ward of an ordinary asylum has not yet been demonstrated. We prefer to allow the *British Medical Journal* and the *Journal of Mental Science* to speak on this subject. The former says, when referring to the proposal of a medical superintendent to establish these hospitals: "We think he will discover what many have discovered before him, that an enormous proportion of cases are associated from the beginning with such a degree of mental degeneration or insane inheritance that anything like 60 per cent. of recoveries is impossible. If it be otherwise, if this high percentage is attainable, we are strongly of opinion that there is no good reason why it should not be attained in existing well-constructed asylums, officered as they are by properly-qualified medical men. . . . They (the hospitals) exist already in Germany in several university towns, but they end in simply resembling the ward for recent or destructive patients in an English county asylum. We believe the results are no better than they would be under the judicious treatment of the able superintendent of the —— Asylum, under his present conditions." The *Journal of Mental Science* states, when reviewing a similar statement by another superintendent, "All this sounds splendid on paper, but we do not hesitate to say it is an illusion. Grant Dr. —— all he asks for, and he will never cure 60 per cent. of his patients. He may rest satisfied that he is at present curing just as many curable patients as would recover under the circumstances he covets. There is no reason whatever why the conditions present in a good county asylum should prevent patients recovering if they are curable. . . . A certain number of cases will get well in any asylum in England; and a certain number are doomed from the time of admission not to get well. It is surprising that so much optimistic nonsense should be talked and written by the superintendents of some asylums." The above views are presumably those of the editors of the *Journal of Mental Science*, Dr. Hack Tuke and Dr. Savage; and it would be hardly possible to bracket two greater living authorities on questions pertaining to lunacy. At all events if we set on one side the epileptics, the paralytics, the cases of organic dementia, the idiots, the cases where insanity is complicated by incurable thoracic and abdominal diseases, and some other forms which need not be specially referred to here, it would seem clear enough that there is not 60 per cent. left to cure.

One thing is certain, and that is the hereditary nature of the disease: hence, to reduce the amount of insanity in the future the greatest care must be exercised in the present in respect to the marriage of those in whom the taint exists.

Alcoholic intemperance is so common and so well-known a cause that nothing more need be said on the subject. With the disuse of alcoholic beverages will come the diminution of insanity; but, alas, the disuse of alcohol is not in the near future. The average cost per head per week in the county asylums is nearly 8*s.* 7*d.*; in borough asylums it is a little over 9*s.* 10*d.* This difference is accounted for partly by the fact that certain charges on the fabric are included in the borough accounts which are omitted in those of the counties, and partly because the numbers are lower in the boroughs than in the counties. A low rate is not, however, an invariable result of high numbers, but it generally is. Reference to numbers 12 and 13 of these articles will show that the rate in the Middlesex asylums is very high, while in the equally large asylums of Lancashire it is very low. The system of management comes in. On one point medical superintendents are unanimous, and that is the extreme importance of early treatment. If this were recognised and acted on, it would do more than anything else in aid of medical treatment, and in promotion of speedy recovery.

The Lunatic Hospitals contain about 2,000 patients. The majority of the hospitals select their cases; that is, epileptics, paralytics, and some other unfavourable varieties of insanity are admitted sparingly, or not at all. The consequence is that the recovery rate is 46 per cent., and the death rate 7 28. Recent legislation, by creating county councils, will necessitate the transfer of the management of county asylums from the visiting justices to delegates from the new Boards. The Lunacy Bill has not yet passed, and it is greatly to be desired that many changes will be made in it before it becomes law. Some of its provisions are useless, others are cumbrous, while there are omissions of very important things, and none more so than the placing of the pensions' question on a satisfactory footing, Indeed, this question may now be said to encircle the whole of the future welfare of our county asylums.

